# A qualitative human factors investigation of the impact of intraoperative CT-based navigation on interprofessional work in the operating theatre

**DOI:** 10.1016/j.sopen.2026.06.005

**Published:** 2026-06-24

**Authors:** Guillaume Lamé, Elyes Gaoua, Basile Heurtault, Antonia Blanié, Charlie Bouthors, Dan Benhamou

**Affiliations:** a Paris-Saclay University, CentraleSupélec, Industrial Engineering Department, 91190, Gif-sur-Yvette, France; b Simulation Center LabForSIMS, Faculty of Medicine, Paris-Saclay University, 91400, Orsay, France; c Paris Region University Hospital, Bicêtre hospital, Department of Anesthesiology-Resuscitation-Perioperative medicine, 94270, Le Kremlin Bicêtre, France; d Paris-Saclay University, Paris Region University Hospital, Bicêtre hospital, Department of Orthopaedic and Traumatologic Surgery, 94270, Le Kremlin Bicêtre, France

**Keywords:** Intraoperative CT, Human factors, Interprofessional teamwork, Ergonomics

## Abstract

**Objectives:**

Intraoperative CT-based navigation (iCT) is increasingly used in complex spine surgeries. We examined the impact of this technology on interprofessional work in the operating room (OR).

**Methods:**

Single-site qualitative study. We interviewed surgeons, anesthesiologists, and nurses involved in spine surgeries with iCT. We modeled the work system using the SEIPS framework and conducted inductive content analysis.

**Results:**

We interviewed 20 professionals. All participants agreed on the benefits of iCT for surgical outcomes. However, it came with ergonomic challenges. The imaging system is a large piece of equipment. Its installation in a preexisting OR resulted in limitations of movements around the patient and in the room. Anesthesiologic and surgical teams required additional time to discuss patient positioning and ensure sufficient space for everyone, as well as adequate access to airways for anesthesiologists and anesthesia nurses. With the new technology also came new tasks. Surgeons usually managed the initial image acquisition, but to avoid desterilization during the procedure, nurses also had to learn to use the equipment. Permanent staff members progressively developed expertise; however, with high turnover among nurses and the use of agency staff, it proved difficult to ensure that surgical nurses were consistently trained in the use of the equipment. Over time, anesthetic and surgical teams adjusted their practices to balance the surgical benefits of the technology with ergonomic concerns.

**Conclusion:**

Implementing iCT can disrupt work systems and requires careful consideration of human and organizational factors in the OR to ensure patient safety. Multicentric studies would be needed.

## Introduction

With the trend toward computer-assisted surgery, work in the operating theatre (OT) has undergone significant changes. For example, the introduction of robotic surgery has modified workflows, workspaces, communication, and coordination between professionals [1]. Intraoperative CT-based navigation (iCT) has received less attention. iCT is particularly used in orthopedic surgery and neurosurgery to assist surgeons in positioning implants, such as deep-brain stimulation implants in neurosurgery [2], or pedicle screws for posterior cervical, thoracic, and long-segment fusions in orthopedic surgery [3].

Research on iCT has focused on clinical outcomes, e.g., precision of screw positioning or risk of bleeding, on surgery duration, and on surgeons' musculoskeletal disorders [4], [5], [6], [7], [8]. The impact on communication, coordination, and workflows remains unclear, although these factors significantly affect operating team performance, professionals' quality of life, and patients' outcomes [9], [10].

We analyzed the impact of iCT on surgical teamwork in the OT. We took a systemic perspective, informed by human factors/ergonomics, to explore how the configuration of tasks, people, spaces, equipment, routines, and their interactions evolved with the introduction of iCT.

## Methods

**Fig. 1:**
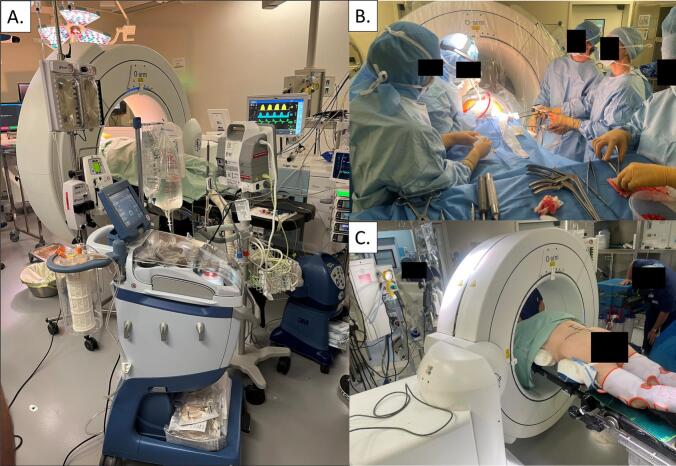
Pictures of the operating theatre with the O-arm™ installed, on the anesthesiologists' side (A), on the surgical side during the surgery (B), on the surgical side before the beginning of the surgery (C).

**Fig. 2:**
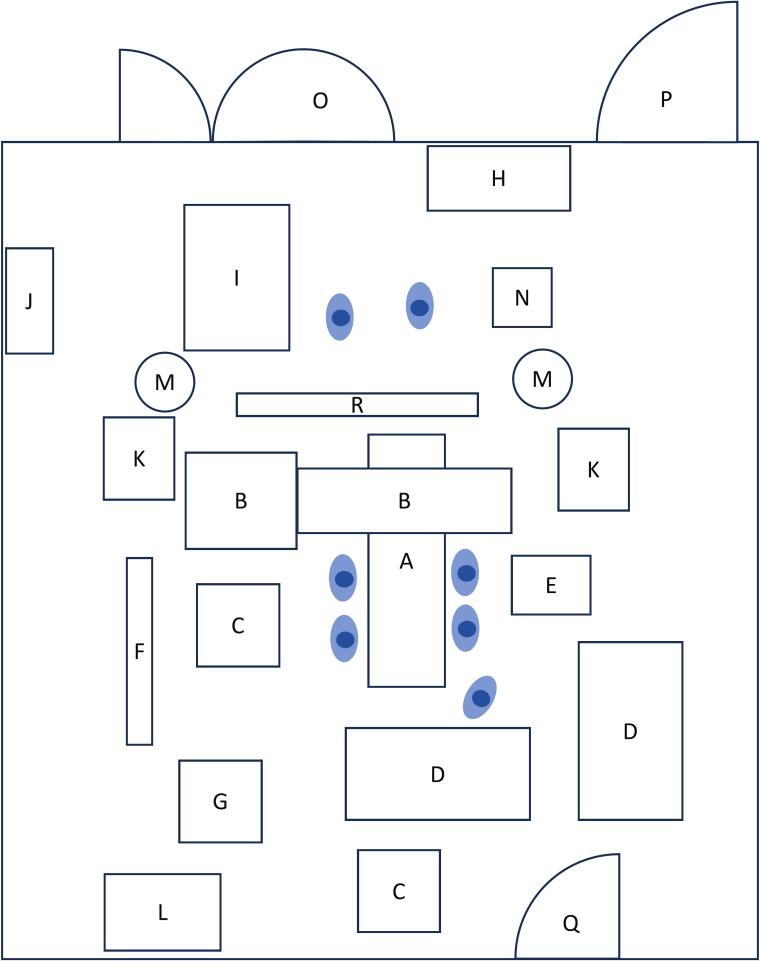
Layout of the operating theatre. Operating table (A), O-arm™ (B), Navigation monitor (C), instrument table (D), electrosurgical unit (E), leaded protection panel (F), O-arm™ computer (G), anesthesia trolley (H), anesthesia workstation (I), room air filtration system (J), distribution arm (K), surgical nurse workstation (L), syringe pole (M), temperature management unit (N), door to induction area (O), door to cleaning area (P), door to surgical storage area (Q), sterile surgical drapes (R).

Between October 2024 and April 2025, we conducted a qualitative, monocentric study through semi-structured interviews. The hospital we studied installed an O-arm™ (Medtronic plc) iCT in an existing OT in 2019 (Fig. 1). The iCT is always installed in an operating room (OR) measuring 44.7 m^2^; it is used for orthopedic surgery and neurosurgery (Fig. 2).

To obtain a 3D reconstruction, the arm must be placed around the patient, forming an O shape. Surgeons have two options: 1. Docking the O-arm™ draped around the patient during surgery imposes moving the ring up toward the anesthesiologist to leave space for the surgical field, or 2. Bringing the O-arm™ only for image acquisition, which leaves more room for the operating field but requires further manipulation by the surgeon or the circulating nurse during the procedure.

We contacted a purposive sample of surgeons, anesthesiologists, anesthetic and surgical nurses, and residents via email and text messages, with at least six months of experience in iCT at the study site. Contacts were suggested by the fourth, fifth and last author based on their experience with iCT. The interview guide was elaborated collectively. Interviews were conducted via videoconference or telephone by the three first authors (all engineers with no medical training), following informed consent. Interviews were audio recorded, transcribed verbatim, and anonymized.

We first mapped the findings to the Systems Engineering Initiative for Patient Safety (SEIPS) 2.0 framework, a human factors model that identifies the main elements interacting in sociotechnical systems in healthcare [11]. In parallel, we performed inductive content analysis [12]. The SEIPS was used as a sensitizing concept in this analysis, drawing attention to important features [13], but without exclusivity or the need to map observations to it. The second and third co-authors read through the transcripts and coded them line by line. The first author checked the coding of all transcripts independently. The three first co-authors then generated higher-level themes. The whole team discussed these themes collectively until we agreed on a final set. The coding process was supported by NVivo (Windows Release 1.7.2, Lumivero, 2024).

We followed the ‘Standards for Reporting Qualitative Research’ checklist [14]. This study was approved by the Institutional Review Board of the French Society of Orthopedic Surgery (SOFCOT) on 11 November 2024 (#28–2024).

## Results

**Fig. 3:**
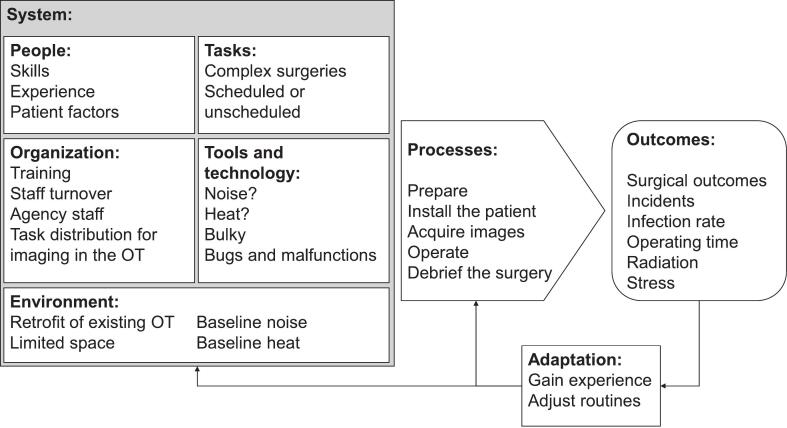
Factors affecting spine surgery with interoperative CT-scan, following the SEIPS framework [11], [32].

We contacted 44 people, of whom 18 never answered despite our chasing (including eleven surgery residents who had moved on to their next internship). Six interviews were canceled and could not be rescheduled within the study timeframe (including four with residents who had moved to other hospitals). We interviewed 20 professionals, covering a balanced sample of surgeons and surgical residents, anesthesiologists, and surgical and anesthesiology nurses of different levels of experience (Table 1). We stopped recruitment due to a combination of time constraints and having contacted our list of potential interviewees, but only two new codes (out of a total of 40) emerged in the last five interviews, suggesting we had reached information saturation. Based on the SEIPS 2.0 modelling (Fig. 3), all elements of the work system were important in the deployment of iCT: tasks (new or reallocated), people (surgeons, anesthesiologists, and surgical and anesthetic nurses), the internal environment (with the iCT installed in a retrofitted room), tools and technologies (the iCT itself), and the organization (training and staffing).

**Table 1:** Characteristics of participants.

	N (%)
Women - Men	9–11 (45–55)
Surgery nurses	4 (20)
Anesthesia nurses	3 (15)
Surgeons	9 (45)
incl. Residents	4 (20)
Anesthesiologists	4 (20)
Interview duration (mean +/− s.d., in minutes)	21.4 +/− 10.4

### Physical impact

The iCT was installed in an existing room that had been specially equipped with lead-lined walls and a reinforced floor. However, the OT was built in the 1980s, and although the room is among the largest in the OT, it remains small to accommodate a scanner.

In spine surgeries, the iCT is used in conjunction with an extensive panel of equipment: specific navigated and non-navigated instruments, iCT, navigation unit, lead glass panel that protects personnel during image acquisition, trolleys for the sensors, and multiple anesthetic modules (e.g., anesthetic station, cell salvage, transfusion, warming system…). As a result, space is very constrained. Circulating around is difficult for the circulating nurse, and the risk of breaching sterility by contact with equipment is higher. Closer to the patient, the surgeons' movements are constrained because the scanner's ring cannot always be removed.

Prior to every operation, anesthesiologists and surgeons must negotiate their space. In some instances, anesthesiologists must relocate some of their equipment in the preparation room. This means a greater flow between the operating room and the external environment, which threatens sterility. Participants underlined that the negotiation was amicable and professional, but everyone was aware of the need to defend their space when planning the surgery or faced issues later on.

*We try to eat into the anesthesiologists' space as much as possible. […] Since we know we're going to have a lot of equipment on our side, that it might be difficult to move around, we try to have as much space as possible. (P1, resident in orthopedic surgery).*

*So when we haven't organized ourselves properly beforehand, when we've tried to be too nice with the anesthesiologists by giving them a lot of space, we have much less, and sometimes we have to kneel to move around the room. (P11, surgical nurse).*

Due to its volume, the scanner also creates a visual barrier between anesthesiologists and surgeons. This point, however, was debated: some on the anesthetic team argued that since transparent sterile covers were used to protect the scanner, rather than the usual opaque blue drapes, they could see better what was happening on the other side.

Some interviewees reported that the scanner generated both noise and heat, but others disagreed, citing the high baseline noise and temperature in the OT.

### New tasks

The iCT creates new tasks and redistributes existing ones. Before surgery, the anesthesiology team gives extra attention to the necessary equipment and patient's positioning. When installing the patient, spine surgery requires prone positioning, necessitating extra care to guarantee access to intravenous lines on the patient's arms. This would also be true without the scanner, but the anesthesiology team found this task more difficult due to the barrier created by the large obstacle installed between them and the surgical team.

Both scrub and anesthesiology nurses reported that surgeons occasionally requested the use of the scanner for emergency surgeries, e.g., on weekends. However, since the O-arm™ is very heavy, the floor in the OR where it has been installed has been reinforced to fit it, but it cannot be moved to another room. Because the scanner is not located in one of the emergency theatres, using it for emergencies meant preparing and opening the iCT room specifically for the use of this equipment. This added to the existing workload.

*When the* O-arm™ *arrived, the surgeons presented the machine and said that it would only be used for scheduled surgeries, not at night or on weekends for emergencies, and so on. But of course, surgeons want to use it on weekends and at night too. […] but they don't necessarily realize the overall impact on our job. We're also convinced of the benefits of* O-arm™ *for patients. But in terms of organization, on weekends or at night, when you have to open an extra room, it creates additional workload. (P17, anesthetic nurse).*

### Technical skills, training, and transfer of knowledge

Interviewees noted that in other hospitals where they had worked, technicians would handle the scan. At the study site, the surgical team must manage data acquisition because there are not enough imaging technicians in the hospital and because data acquisition with the O-arm™ requires specific device positioning, knob knowledge, and imaging competency. In practice, both surgeons and surgical nurses must learn how to use the system and how to troubleshoot it when necessary. Surgeons are more expert in this task, and they can handle the initial image acquisition, but once they are in sterile gowns, they must rely on nurses to navigate the system during the operation. This usually works smoothly with experienced nurses; however, given the high turnover in the OT, surgeons often must work with agency staff or newcomers who are unfamiliar with the system. Experienced permanent staff often need to introduce temporary workers to the machine.

*There's a serious shortage of staff. That means we regularly work with temporary staff or people who don't know much, if at all, about the* O-arm™ *. So, when we work with these people, who often adapt quickly, that's not the problem, but we inevitably end up re-explaining the* O-arm™ *every time, quickly going through the essentials, and then we just must make it work*. (P10, surgical nurse).

However, even having trained permanent nursing staff is challenging, given the high rate of staff turnover. Some surgeons tend to do everything themselves, and surgical nurses do not get the opportunity to practice.

Training is on-the-job and through experience; many respondents reported that they had not received formal training. The equipment manufacturer delivered training sessions after installation, but only some of our participants attended. The hospital operates a simulation training program, including in situ sessions in the OT, but the O-arm™ has not yet been included in a scenario. Some residents discovered the equipment on the spot.

*I didn't know what it was. They told me, ‘Yes, there's an* O-arm™’*, but I didn't understand what that meant. I didn't know how to spell it. I arrived, saw a big scanner, and said, ‘OK, that's it.’ Then they told me, ‘Here, it works like this, like this, if you ever need it.’ And then you look and try to understand, and eventually you get there. But it would be nice if there was someone who specialized in it and could train us on how to do it. (P9, intern in orthopedic surgery).*

### Benefits and risks

Interviewees all considered that the iCT enabled more precise implant positioning and improved surgical quality. Some surgeons said that this had been established in the literature. The iCT does not enable new surgeries to be performed but provides safety guarantees, particularly for surgeries of the higher thoracic and lower cervical spine, where traditional imaging methods are less practical and effective. Participants were uncertain about whether the iCT reduced operating time. They said that the surgery preparation took longer, but that the operation itself might be shortened.

One interviewee also noted that, as this technology is still available only in a handful of training hospitals, having iCT helped attract residents. In practice, some residents said that they actually got less responsibility in the surgery because the main surgeon would want to operate the scanner themselves.

*Maybe the consultants do more. Especially since it's a bit of a new gadget, there are often two consultants and one intern. Whereas usually it's just one chef and one intern. So we have less to do* (P11, orthopedic surgeon resident).

In contrast, senior surgeons noted that the navigation system can aid them in supervising trainees during procedures, as it displays real-time images of the instrumentation during placement, thereby facilitating surgical training and knowledge transfer.

*Being able to more easily help residents, who learn with me. Because I can see precisely what they're doing when they are working on the vertebra, I see exactly what direction the instrument is taking. Whereas when it's only by hand, I can't see. I can estimate, but I'm not sure, whereas here I have a very precise view of what they're doing. This is one of the big, big advantages of the* O-arm™ *.* (P4, senior orthopedic surgeon).

Surgeons mentioned the risk of ‘drift’, or losing accuracy, when there is a mismatch between the navigation system and the patient's reference frame, or when the reference frame is accidentally moved during surgery.

If everyone acknowledged the benefits for the patient and for surgical practice, anesthesiologists and anesthetic nurses also clearly stated that the scanner had no direct benefit for them. In fact, it made it more difficult to respond to some emergencies, where quick access to the patient's airways is critical. This is particularly true for cervical surgeries, where the head of the patient is inside the scanner's hoop.

*For some major surgeries like vertebrectomies, the patient is in prone position with a double-lumen tube, something that's quite risky, where we want to have access to the patient's head, and those are surgeries with a lot of bleeding. So there's also a cell saver, there's a lot of equipment and monitoring systems,* etc. *So it's sort of housing crisis. Based on ergonomic considerations, to have space and most of all to have access to the patient's head, we sometimes choose not to leave the* O-arm™ *during the whole surgery and to reposition it only at the end to verify screw positions. (P2, anesthesiologist).*

Due to the crowded space in the OT, many interviewees feared sterility issues from contact with the iCT. Sterility was also threatened by movements in and out of the room during image acquisition.

Finally, many participants expressed concern about radiation, with nurses being the most worried. Surgeons argued that without the scanner, they would use other imaging techniques that would also generate radiation.

### Adaptation and collective learning

Integrating the iCT into an existing environment was a learning process, both at the individual and collective levels.

Individually, surgeons mentioned that they became much quicker at navigating the scanner with experience. Surgery nurses also learned to support surgeons with navigation during operations, and both learned their way around the system's frequent bugs. In the early days, biomedical engineers or the iCT manufacturer's technicians were often called, but this need decreased over time with experience. As time passed, some professionals in the OT developed expertise and became go-to persons for issues with the iCT.

*With experience, it's less complicated. It's true, sometimes they call me in the room because it doesn't work. So in general I'll solve the issue and, and it works fine. (P7, surgical nurse).*

Collectively, the surgical and anesthesiology teams negotiated new arrangements. They acquired a new operating table to enhance patient arm support. They decided to change the patient's orientation in the room for certain procedures, allowing the anesthesiology team to more easily access the patient's airways. Anesthesiologists decided to prospectively add a second peripheral vein catheter for some surgeries with difficult access, in case they needed it. For some surgeries, they convinced surgeons that the O-arm™ needed to be removed after image acquisition was complete. Rules established in the early days, like “no more than two procedures with this technology per staff member per week”, were progressively lifted.

*Once or twice we've had issues accessing patients' airways, and that's why, I told you, we've got more experience, we have more specific requests and we say for the next similar surgery, we won't accept having the* O-arm™ *[…] In fact, we've requested that there is, for example, three image acquisitions, one before, one during or more during the surgery and one after, and in between, they remove the hoop* (P3, anesthesiologist).

However, this learning remained informal. Some participants mentioned that they had written a leaflet, procedure, or guidebook on working with the scanner, but when prompted about guidelines, no participant was aware of an existing document.

Some participants regretted the lack of collective discussion about how best to adapt to the new technology. Complex surgeries are discussed in medical staff meetings, but among anesthesiologists, the conversation was sometimes difficult because some anesthesiologists had never worked with it and could therefore not understand the challenges. Between anesthesiologists and surgeons, there was no established interdisciplinary routine for preparing and debriefing complex surgeries involving iCT. At the inter-organizational level, conversations were also hampered by the limited diffusion of the technology, with only a few centers equipped.

## Discussion

New technologies are not plug-and-play; they must fit within an existing system and will often disrupt it. In this single-center study, the implementation of a new iCT in the OT affected many aspects of surgical work. Installing a large device in the existing environment meant less space was available, affecting movements on and around the operating table. Surgeries that were already complex required even more planning, a point particularly mentioned by anesthesiologists. Surgeons and nurses learnt how to operate the equipment on the job, in an ad-hoc fashion. Progressively, everyone improved their skills and learnt how to circumvent the frequent technical issues. However, this learning process was hindered by the high staff turnover and reliance on temporary staff in the OT, particularly among surgical nurses.

This situation is not unfamiliar to HF/E observers. Retrofitting an existing OT to accommodate new equipment is a highly constrained operation: as little as possible must be changed to avoid running over budget, and staff must become creative to make things work [15]. Staffing is a recurring issue in hospitals, which undermines everything else. If the team is already stretched, it becomes more challenging to allocate time for training or engage in collective discussions about new routines. In this situation, under both workload and economic pressures, it takes real effort to keep within the margin of a safe operation [16]. In particular, it requires that everyone focus on the ultimate goal, even when the operational benefits of the new system are unevenly distributed. Here, anesthesiologists faced additional constraints but saw no improvement in their own work from the iCT; instead, they experienced mainly additional constraints. They had to defend their perspective, in particular their need to access the patient's airways in case anything untoward happens. Even inside the anesthesiology department, not all practitioners took part in iCT surgeries, showing how the constraints were born by a minority. Nurse anesthetists often shared this view. Although they recognised the added value of using iCT, anesthesia providers felt that their constraints were not being taken into account and that their negative views were perceived as those of ‘troublemakers’ keen to delay the implementation of modern techniques, thereby acting against patients' needs. Such ‘micropolitics’ are essential to the success of the implementation [17]. In our case, new routines were negotiated without entirely erasing tensions around the use of space in the OT. Yet, these adaptations demonstrate the benefit of ‘double loop learning’, whereby staff address ‘root causes’ to prevent problems from recurring [18].

Our results echo previous findings. A recent systematic review examined the impact of navigation on processes and outcome indicators [6]. Radiation risks appeared lower with navigation than with previous techniques, and the accuracy of pedicle screw positioning improved. The impact on operating time was unclear. A previous meta-analysis found a significantly longer operating time, but fewer complications, in navigation-assisted surgery. This somehow mirrors our interviewees' answers: better surgical quality, uncertain process impact. The drift phenomenon mentioned by some of our participants appeared in one-sixth of the cases (16.7%) in Zhao et al.'s cohort study [19]. Finally, a ‘learning curve’ has also been evidenced, with around 30–35 surgeries or 200 pedicle screw placements before reaching a performance plateau [8]. Our study shows that this learning curve is fundamentally a collective phenomenon: because the new technology creates new tasks and redistributes or reconfigures others, the multidisciplinary team as a whole must progress, not just the surgeon. In addition, in academic institutions, the operating team is almost never the same, and learning should be distributed to a large number of personnel to ensure that, regardless of the crew on board, everybody is accustomed to the device and the subsequent organization. Finally, previous research has shown how novice surgeons exhibit significantly greater neurophysiological and endocrine stress responses during surgeries [20], a phenomenon to which new technologies, discovered on the spot with little training, may contribute.

Facing similar situations, most at-risk industries mandate preliminary training of professionals when deploying new equipment (c.f. French regulation in commercial aviation [21]). Recent French guidelines promote the same approach in OTs [22]. In particular, simulation training has the advantage of teaching both technical and non-technical skills in contexts such as robotic surgery. It can cover both regular and crisis situations [23]. Yet, our results show that training remains difficult to deliver consistently over time. We need strategies to ensure that all users are trained when new equipment is installed, and that new staff members receive adequate training on equipment that is not standard practice. Implementation science could help design and tailor these strategies to different contexts [24]. Finally, using technology to attract residents and physicians aligns with the idea that a key driver for medical students is to gain clinical experience through their training [25], [26].

This is the first study to demonstrate how the work system is reconfigured following the introduction of iCT. The effect of workload and staffing on care quality is established [27], but the issue extends beyond having enough staff. Stability is also important, as it allows people to master rare techniques that they would not acquire in most other centers. Practical issues, such as room size, may not be the primary concern when investing in innovative technologies, but they create significant problems on the frontline. Ultimately, distractions and interruptions are detrimental to the smooth process of surgeries [28]; poor layout and crowded spaces affect postures, generate hazards, and undermine staff wellbeing [29], [30].

This study has limitations. Because it is a single-site case study, some of the results may be attributed to the specific configuration of the OT we studied: no imaging technicians in the OT, a small room that increased the ‘crowding’ effect, only one room where the iCT could be used, so it had to be opened specifically for out-of-hours emergency surgeries. Together, these factors create a configuration that may not be typical. Indeed, our informal contacts with surgery professionals in other hospitals suggest that having imaging technicians or setting up the O-arm in larger, more recent OTs relieves some issues. Architectural conditions (short distance between ORs to reduce the difficulty of moving the iCT; a greater number of ORs designed to provide protection against X-rays; and a more robust floor to support the weight of the iCT) may also facilitate the use of the iCT in emergency conditions and in a larger number of ORs. On the other hand, personnel shortages, staff turnover and the installation of new technologies in existing environments are common in healthcare systems.

Interviews provided us with insights into participants' subjective views, but a multisite study, combining interviews with observations (in real or simulated situations) [31], would be beneficial. Indeed, our conclusions about disruption, adaptation, and risk are based entirely on participant perceptions, which are important but not the same as direct evidence of workflow consequences. Observations are invaluable to appreciate ‘work as done’, which may help contextualize the perception of those involved.

## Conclusion

Adding new technologies to existing environments requires careful adaptation and sustained effort to train staff and adjust practices. Staffing issues, practical constraints, and individual and collective learning interact in these projects. To maximize the benefits of new technologies, hospitals must integrate these factors into their acquisition and implementation plans. Yet, these are findings from a single centre study, so larger studies would help identify what factors, and combinations of factors, are particularly influent.

## CRediT authorship contribution statement

**Guillaume Lamé:** Writing – review & editing, Writing – original draft, Visualization, Supervision, Software, Project administration, Methodology, Investigation, Formal analysis, Data curation, Conceptualization. **Elyes Gaoua:** Writing – original draft, Investigation, Formal analysis, Data curation. **Basile Heurtault:** Writing – original draft, Methodology, Investigation, Formal analysis, Data curation. **Antonia Blanié:** Writing – review & editing, Supervision, Project administration, Methodology, Conceptualization. **Charlie Bouthors:** Writing – review & editing, Supervision, Project administration, Methodology, Conceptualization. **Dan Benhamou:** Writing – review & editing, Supervision, Project administration, Methodology, Conceptualization.

## Ethics and other permissions

Approved by the Institutional Review Board of the Société Française de Chirurgie Orthopédique et Traumatologique (SOFCOT) on 11 November 2024 (ref 28–2024)

## Funding

This study did not benefit from specific funding.

## Declaration of competing interest

The authors declare that they have no known competing financial interests or personal relationships that could have appeared to influence the work reported in this paper.
